# Fluorescein Leakage within Recent Subretinal Hemorrhage in Pathologic Myopia: Suggestive of CNV?

**DOI:** 10.1155/2018/4707832

**Published:** 2018-08-13

**Authors:** Lan Mi, Chengguo Zuo, Xiongze Zhang, Bing Liu, Yuting Peng, Feng Wen

**Affiliations:** ^1^State Key Laboratory of Ophthalmology, Zhongshan Ophthalmic Center, Sun Yat-sen University, Guangzhou 510060, China; ^2^Department of Ophthalmology, Guangdong Provincial Hospital of Traditional Chinese Medicine, Guangzhou 510060, China

## Abstract

**Purpose:**

To determine whether fluorescein leakage within subretinal hemorrhage is definitely suggestive of choroidal neovascularization (CNV) by multimodal imaging including optical coherence tomography angiography (OCTA).

**Methods:**

Twenty-five consecutive highly myopic patients (25 eyes) with fluorescein leakage within subretinal hemorrhage detected within 1 month were prospectively included. All patients underwent OCTA and spectral-domain optical coherence tomography (SD-OCT). The OCTA and SD-OCT findings at the site of fluorescein leakage were analyzed. In cases of a doubtful diagnosis, indocyanine green angiography (ICGA) was also performed to differentiate myopic CNV from lacquer crack if necessary; all patients were followed up by SD-OCT and/or OCTA for at least 2 weeks.

**Results:**

In terms of the site of fluorescein leakage, OCTA revealed an abnormal vascular network in the outer retina and a choriocapillaris slab in 22 out of 25 eyes (88%), which were confirmed to be CNV. However, no high-flow signal was observed in 3 of 25 eyes (12%). In these 3 cases, SD-OCT showed a focal rupture of the retinal pigment epithelium-Bruch's membrane-choriocapillaris (RPE-BM-CC) complex and a columnar hyperreflective signal of blood originating from defects with a volcanic geyser-like appearance, and no exudative signs were detected. Notably, all ruptures of the RPE-BM-CC complex were located exactly at lacquer crack sites. Moreover, with the absorption of subretinal hemorrhage, ruptures of the RPE-BM-CC complex spontaneously resolved without any intervention. Considering the multimodal imaging appearance and follow-up outcomes, these 3 eyes were eventually diagnosed as simple bleeding associated with lacquer cracks.

**Conclusions:**

Dye leakage within recent subretinal hemorrhage on FA could be caused by new-onset lacquer cracks in pathologic myopia. Multimodal imaging including OCTA is helpful to differentiate lacquer cracks from myopic CNV.

## 1. Introduction

Pathologic myopia is defined by an axial length of the eye greater than 26 mm or a refractive error of at least −6 diopters (D) and is associated with complications of the posterior segment secondary to excessive elongation of axial length. Myopic maculopathy, such as diffuse and patchy chorioretinal atrophy, lacquer cracks, myopic choroidal neovascularization (CNV), myopic subretinal hemorrhage (mSH), and posterior staphyloma, has been reported to be a significant cause of visual impairment and legal blindness worldwide, especially in Asian countries [1–4]. Subretinal hemorrhage is frequently seen with recent myopic CNV but is also a common finding in cases of new-onset lacquer cracks [5–7]. Myopic CNV is the most common central vision-threatening complication in patients with high myopia, affecting 5% to 11% of cases [8]. Once confirmed as active CNV, timely treatment such as anti-VEGF therapy is recommended. However, mSH associated with lacquer cracks, which is also called simple bleeding, generally has a better prognosis. Therefore, the differentiation and diagnosis of these two types of subretinal hemorrhage are crucial.

Fluorescein angiography (FA) is an essential method for the diagnosis of myopic CNV. Recent myopic CNV usually displays well-defined hyperfluorescence in the early phase with dye leakage in the late phase [5]. However, simple bleeding associated with lacquer cracks is generally thought to display blocked fluorescence. In our clinical work, an incidental case of pathologic myopia with new-onset extrafoveal hemorrhage attracted our attention (Supplementary Materials (available here), which illustrates this case; https://figshare.com/s/7dcf53004450e72f209b). SD-OCT revealed a focal rupture of the retinal pigment epithelium-Bruch's membrane-choriocapillaris (RPE-BM-CC) complex underlying the hemorrhage without an exudative sign, which corresponded to the site of lacquer crack confirmed by indocyanine green angiography (ICGA), while FA presented mild fluorescein leakage within the hemorrhage. Notably, with the absorption of subretinal hemorrhage, the ruptured RPE-BM-CC complex completely resolved spontaneously. In view of multimodal imaging features and outcome, this case was presumed to be simple hemorrhage associated with lacquer crack. We were curious about fluorescein leakage caused by lacquer cracks. As proposed by Klein and Curtin [9], if lacquer cracks are indeed defects in the RPE-BM-CC complex, leakage of fluorescein from the choriocapillaris may be expected. Indeed, previous studies have reported discontinuities in the RPE-BM-CC complex at the site of lacquer cracks on spectral domain-optical coherence tomography (SD-OCT) [10, 11]; however, until very recently, no previous studies have reported dye leakage associated with lacquer cracks on FA.

The primary purpose of this study was to investigate whether fluorescein leakage within recent subretinal hemorrhage in pathologic myopia is suggestive of CNV.

## 2. Methods

This is a prospective observational case series of patients from the fundus disease clinic of the Zhongshan Ophthalmic Center who presented between November 2016 and December 2017. The protocol of this study was approved by the Ethics Committee of the Zhongshan Ophthalmic Center of Sun Yat-sen University, and the study was performed in accordance with the Declaration of Helsinki.

The inclusion criteria were as follows: (1) high myopia (refractive error ≤ −6 D or axial length ≥26 mm); (2) within 1 month after the onset of ophthalmic symptoms including acute visual loss and fixed scotoma with or without metamorphopsia; and (3) dye leakage within subretinal hemorrhage on FA. Fluorescein leakage was characterized by increased size and intensity.

The exclusion criteria were as follows: (1) mild or moderate myopia (refractive error ≥ −6 D); (2) clinical features of age-related maculopathy such as soft or hard drusen; (3) a history of other ocular disorders or intraocular surgery, such as multifocal choroiditis or punctate inner choroidopathy, ocular trauma, severe cataract, retinal artery or retinal vein occlusion, diabetic retinopathy, or other retinal vascular diseases; and (4) previous ocular therapy, such as laser photocoagulation, photodynamic therapy, or intravitreal injection of anti-VEGF.

Each patient underwent a comprehensive ophthalmologic examination, including assessment of the best-corrected visual acuity (BCVA) using Snellen charts, dilated slit-lamp anterior segment and fundus biomicroscopy, fundus photography, OCTA, and SD-OCT. The diagnosis of CNV based on OCTA was defined as a vascular network pattern in outer retina and choriocapillaris slab corresponding to the site of fluorescein leakage [5]. The diagnosis of CNV based on SD-OCT was defined as hyperreflective lesion contiguous above the RPE, with or without an associated exudative sign, such as subretinal fluid, intraretinal fluid, and subretinal hyperreflective exudation [5]. Corresponding to the leakage site on FA, patients with neither abnormal high-flow vascular network on OCTA nor CNV appearance on SD-OCT were rated as a doubtful case, for which, ICGA was also performed. Furthermore, SD-OCT and OCTA were performed again within 2 weeks after the initial clinical examination.

Photographic images of the fundus were obtained using confocal laser scan systems (Carl Zeiss, Inc., Jena, Germany). FA and ICGA images were obtained using confocal laser scanning systems (Spectralis, Heidelberg Engineering, Dossenheim, Germany). SD-OCT was performed with an HRA + OCT Spectralis (Spectralis, Heidelberg Engineering, Dossenheim, Germany). Horizontal and vertical SD-OCT scans of 6 mm or 3 mm were centered on the lesions with dye leakage on FA. OCTA images were obtained using the AngioVue imaging system (Optovue Inc., CA, US) with the macular cube (3 × 3 mm and/or HD 6 × 6 mm) protocol. Each OCTA volume involved in the bleeding area contained 304 × 304 A-scans with 2 consecutive B-scans. Automatic retinal segmentation was performed by embedded software in the machine. Outer retina slab is autosegmented from 9 *μ*m beneath the OPL to 9 *μ*m above the BRM and choriocapillaris slab is autosegmented from 9 *μ*m above the BRM to 31 *μ*m beneath the BRM. Two ophthalmologists (LM and CGZ) independently assessed the multimodal images.

## 3. Results

Twenty-five highly myopic eyes (25 patients) were included in this study. The subjects included 10 men and 15 women with a mean age of 37.8 ± 12.1 years (range: 21–56 years). The mean refractive error was −12.2 ± 3.2 D (range: −7 D to −19.0 D). The mean baseline BCVA was 0.34 ± 0.22 (range: 0.1–1.0). The mean duration of visual symptom was 16.9 ± 6.8 days (range: 5–29 days). Demographic and morphologic characteristics of the study population are listed in Table 1. Relevant data have been uploaded to figshare, the link is https://figshare.com/s/dc2237097539fcdeff4e.

### 3.1. FA and SD-OCT Findings

Blocked fluorescein from subretinal hemorrhage was observed in all cases. Of these, 21 hemorrhages covered the central fovea. Well-defined hyperfluorescence during the early phase with fluorescein leakage during the late phase was detected in the bleeding areas. All patients were preliminarily diagnosed with myopic CNV based on the FA appearance. Exudative signs corresponding to the site of fluorescein leakage were observed on SD-OCT in 22 out of 25 eyes (88%). Of these, SD-OCT revealed intraretinal cysts in 4 of 25 eyes (16%), subretinal fluid in 10 of 25 eyes (40%), and subretinal hyperreflective exudation in 17 of 25 eyes (68%).

### 3.2. OCTA Findings

**Figure 1:**
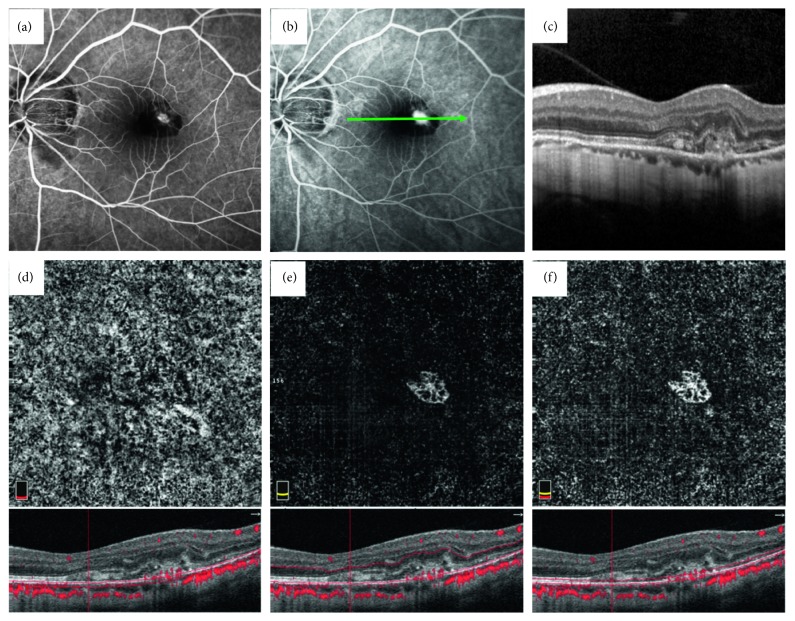
Myopic CNV with recent subretinal hemorrhage imaged by FA, SD-OCT, and OCTA. Left eye imaging of a 44-year-old man preliminarily diagnosed with myopic CNV by FA (refractive error: −9.0 D). Fluorescein angiography showing early hyperfluorescence (a) with late leakage (b). Spectral-domain optical coherence tomography scan passing through the site of leakage revealing CNV, as evidenced by a hyperreflective lesion with the RPE with subretinal hemorrhage and slight subretinal fluid (c). Optical coherence tomography angiography 3 × 3 mm images including the choriocapillaris slab (d), the outer retinal slab (e), and the RPE slab (f). Abnormal vascular network is less visible on the choriocapillaris slab (d) and is best visible on the RPE slab (f).

OCTA revealed an abnormal vascular network in the outer retina and the choriocapillaris slab in 88% of eyes (22 of 25), whereas no high-flow signal was observed in 12% of eyes (3 of 25). Those 22 eyes with classic vascular network on OCTA were confirmed to be CNV. The lesion was visible on the outer retinal slab and less visible on the choriocapillaris slab, and the best visualization of the CNV network corresponded to a manual segmentation mostly on the RPE (a 40 *μ*m manual segmentation was implemented, and the lower boundary of this manual segmentation was localized on BM). A representative case is described in Figure 1.

**Figure 2:**
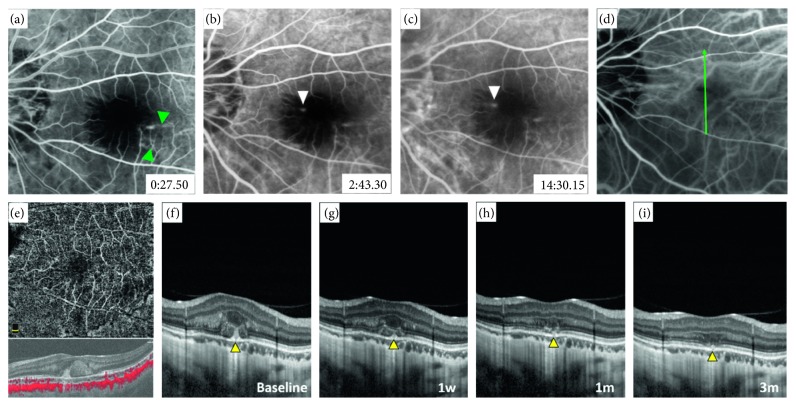
Multimodal imaging findings in a 27-year-old girl presenting with new-onset decreased visual acuity and fixed scotoma in front of her left eye for 2 weeks (refractive error: −19.0 D). At the time of 27.50″ after dye injection, (a) fluorescein angiography (FA) showing blocked fluorescence from subretinal hemorrhage in the macular area and with several linear transmission hyperfluorescence outside it (window defect, green arrowhead). At 2′43.30″, FA revealing focal hyperfluorescence (b) within bleeding area (white arrowhead) (c) and with slight leakage during the late phase (white arrowhead). (d) Indocyanine green angiography revealing linear hypofluorescence of a lacquer crack (green arrowhead) corresponding to the site of fluorescein leakage. (e) Optical coherence tomography angiography showing no high-flow signal in the outer retinal segmentation. (f) Spectral-domain optical coherence tomography revealing focal rupture of the RPE-BM-CC complex with a volcanic geyser-like appearance, without an exudative sign. With the absorption of the hemorrhage, the ruptured RPE-BM-CC complex gradually resolved during the 3-month follow-up (g–i).

**Figure 3 fig3:**
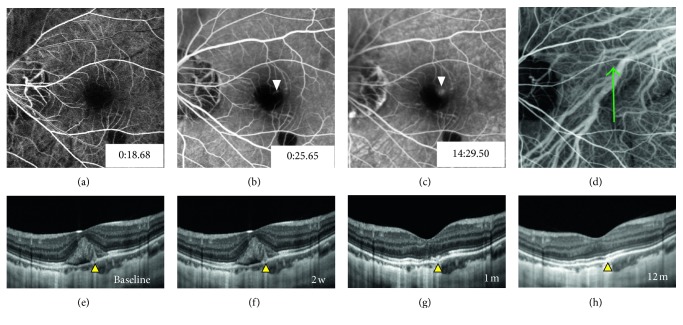
Multimodal imaging characteristics of a 29-year-old girl with acute visual loss and fixed scotoma in the front of her left eye for 1 week (refractive error: –14.0 D). At the time of 18.68 “after dye injection, (a) fluorescein angiography (FA) showing two blocked fluorescence from subretinal hemorrhages in the macular area, one involving central fovea. At 25.65”, FA revealing focal hyperfluorescence, (b) within the bleeding area (white arrowhead), and (c) with mild leakage during the late phase (white arrowhead). (d) Indocyanine green angiography showing linear hypofluorescence of a lacquer crack. (e) Spectral-domain optical coherence tomography showing a focal rupture of the RPE-BM-CC complex with a volcanic geyser-like appearance, without an exudative sign (yellow arrowhead). The ruptured RPE-BM-CC complex gradually resolved during the 12-month follow-up (f–h).

**Figure 4:**
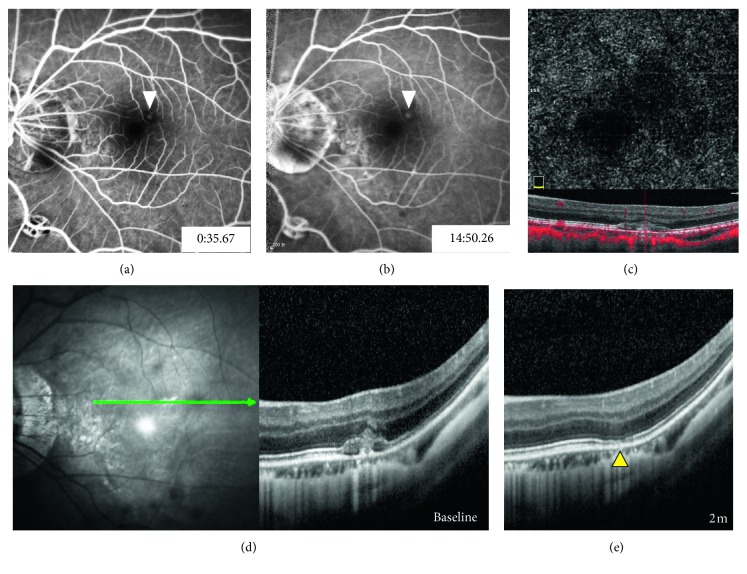
Multimodal imaging findings in a 49-year-old highly myopic man with left eye metamorphopsia for 5 days (refractive error: −13.5 D). At the time of 35.67″ after dye injection, (a) focal hyperfluorescence within extrafoveal bleeding site appearing on FA and (b) with mild fluorescein leakage during the late phase (arrowhead). (c) OCTA showing no abnormal vascular network. (e) SD-OCT showing a focal rupture of the RPE-BM-CC complex corresponding to the site of fluorescein leakage (yellow arrowhead). (e) Continuity of the ruptured RPE-BM-CC complex resolved during a 2-month follow-up (yellow arrowhead).

In the remaining 3 eyes with no high-flow signal on OCTA, SD-OCT revealed subretinal hemorrhage with focal rupture of the RPE-BM-CC complex. A columnar hyperreflective signal of blood originating from ruptures with a volcanic geyser-like appearance was observed. Neither hyperreflective lesion contiguous with the RPE nor exudative signs, such as subretinal fluid, intraretinal cysts, or subretinal hyperreflective fuzzy exudation, were detected. ICGA was also performed and revealed that ruptures of the RPE-BM-CC complex captured via SD-OCT were located exactly at the site of the lacquer crack. The preliminary diagnosis of myopic CNV was disregarded; thus, no interventional therapy was administered. Notably, with the absorption of subretinal hemorrhage, all the ruptured RPE-BM-CC complexes appeared to spontaneously resolve during the follow-up period of 2 to 3 months without any interventions. These 3 leakage lesions were eventually considered to be a result of the rupture of the RPE-BM-CC complex caused by lacquer cracks, and these cases were finally diagnosed as simple hemorrhage. Representative cases are listed in Figures 2 [Fig fig3]–4.

## 4. Discussion

Fluorescein angiography (FA) is an important tool for identifying myopic CNV and for evaluating its activity. Active myopic CNV usually involves well-defined hyperfluorescence during the early phase with dye leakage during the late phase, even in cases with subretinal hemorrhage, because the hemorrhage usually does not completely mask or cover the underlying CNV [5]. Therefore, patients with early hyperfluorescence and late fluorescein leakage within subretinal hemorrhage are usually diagnosed with active CNV and receive intravitreal injection of anti-VEGF in clinical practice. However, this study found that dye leakage within simple bleeding could also be caused by lacquer cracks on FA.

In this study, 25 eyes preliminarily diagnosed as myopic CNV with subretinal hemorrhage by FA were analyzed. Twenty-two of 25 patients (88%) presented with classic CNV network on OCTA. However, the preliminary diagnosis of CNV in the remaining 3 of 25 cases (12%) was finally disregarded. By combining the OCTA, SD-OCT, and ICGA appearance, these 3 cases were eventually diagnosed as simple bleeding associated with lacquer cracks. Moreover, the self-limiting outcomes further support the final diagnosis. This result demonstrated that lacquer cracks within recent myopic subretinal hemorrhage can also cause fluorescein leakage.

To date, no prior study has reported dye leakage associated with lacquer cracks on FA; previous researchers ascribed it to a closed defect caused by an avascular barrier, such as scar tissue. In this study, three patients with fluorescein leakage associated with lacquer cracks were initially examined within 2 weeks after the onset of visual symptoms. Apparent defects of the RPE-BM-CC complex corresponding to lacquer cracks were also detected with SD-OCT. We speculate that fluorescein leakage within simple myopic hemorrhage depends on the extent of the damage to the RPE-BM-CC complex and when the hemorrhage occurred. Perhaps, early-onset hemorrhage associated with lacquer crack combined with a secondary relatively large rupture of the RPE-BM-CC complex possibly results in dye leakage on FA, which may easily be misdiagnosed as myopic CNV and should be noted in clinical practice.

Lacquer cracks, which are a hallmark of pathologic myopia, typically appears as yellowish linear lesions in the posterior pole of the eye and are widely considered to be mechanical breaks in the RPE-BM-CC complex secondary to excessive axial elongation [9]. Lacquer cracks usually present as window defect on FA. Newly developed lacquer cracks may be seen with subretinal hemorrhage [6, 7, 12]. Subretinal bleeding without CNV in pathologic myopia is also considered to be a sign of new lacquer crack formation [7]. As proposed by Klein and Curtin [9], if lacquer cracks are indeed defects in the RPE-BM-CC complex, leakage of fluorescein from the choriocapillaris might be expected. Similarly, it is also speculated that if the defect in the RPE-BM-CC complex caused by a lacquer crack is large enough, detectable rupture of the RPE-BM-CC complex on SD-OCT may be detected. Indeed, Hung et al. and Shinohara et al. reported discontinuities of the RPE-BM-CC complex at the site of lacquer cracks on SD-OCT in some high myopic eyes [10, 11].

The clinical diagnosis of myopic CNV is usually confirmed by FA. Diagnosis features on FA include well-defined hyperfluorescence during the early phase and a classic CNV pattern of leakage (usually mild leakage) [5, 13–15]. Leveziel et al. reported that exudative features of myopic CNV are more obvious on FA than on SD-OCT and suggested that FA should be performed in any case of suspected new-onset myopic CNV [16]. Garcia-Layana et al. reported that the sensitivity of OCT performed after a diagnosis of myopic CNV was confirmed to be 97% for detecting CNV activity during treatment [17]. Indeed, to increase the diagnostic accuracy, it is generally recommended to perform both FA and SD-OCT in doubtful cases [18]. OCTA has proven to be particularly valuable for the diagnosis of myopic CNV with high sensitivity and specificity (>90%) [19–21]. In their study, Miyata et al. reported that there was no false-positive detection of CNV by OCTA in treatment-naive patients and even suggested that, if myopic CNV is detected by OCTA, FFA becomes unnecessary [21]. In this study, multimodal imaging including OCTA, SD-OCT, and ICGA excluded the preliminary diagnosis of myopic CNV in 3 eyes, demonstrating that FA alone might not be the optimal method for initial CNV detection in high myopia with subretinal hemorrhage.

Our study has several limitations that need to be considered. Firstly, the number of patients participated in this study is relatively small. Secondly, all patients were from a single institution; thus, a referral bias may exist. Thirdly, as a clinic-based observational study, selection bias may exist. However, this study is the first to report fluorescein leakage of lacquer cracks, which is easy to be misdiagnosed as active CNV.

In conclusion, we observed a series of highly myopic patients with fluorescein leakage within recent subretinal hemorrhage and found that the ruptured RPE-BM-CC complex caused by lacquer cracks can also present as fluorescein leakage, which needs to be noted in clinical practice, especially in cases with new-onset subretinal hemorrhage.

## Figures and Tables

**Table 1:** Demographic and morphologic characteristics of the study population.

Patient characteristics (*n*=25)	
Age, years (range)	37.8 ± 12.1 (21–56)
Sex (male/female)	10/15
Mean refractive error, diopters (range)	−12.2 ± 3.2 D (−7.0 to −19.0 D)
Right eye (*n*)	15
Left eye (*n*)	10
Best-corrected visual acuity (range)	0.34 ± 0.22 (0.1–1.0)
Duration of visual symptom, days (range)	16.9 ± 6.8 (5–29)

## Data Availability

The data used to support the findings of this study are included within the article.

## Supplementary Materials

Supplementary Materials The supplementary material describes multimodal images of a highly myopic patient with fluorescein leakage caused by lacquer crack within recent subretinal hemorrhage.
