# Supplementary material for: Fluorescein Leakage within Recent Subretinal Hemorrhage in Pathologic Myopia: Suggestive of CNV?
Source: J Ophthalmol. 2018 Aug 13;2018:4707832. doi: 10.1155/2018/4707832 (PMC6110035; doi:10.1155/2018/4707832)
Supplement: Supplementary Materials — The supplementary material describes multimodal images of a highly myopic patient with fluorescein leakage caused by lacquer crack within recent subretinal hemorrhage. [file 4707832.f1.pdf]

Supplementary Materials

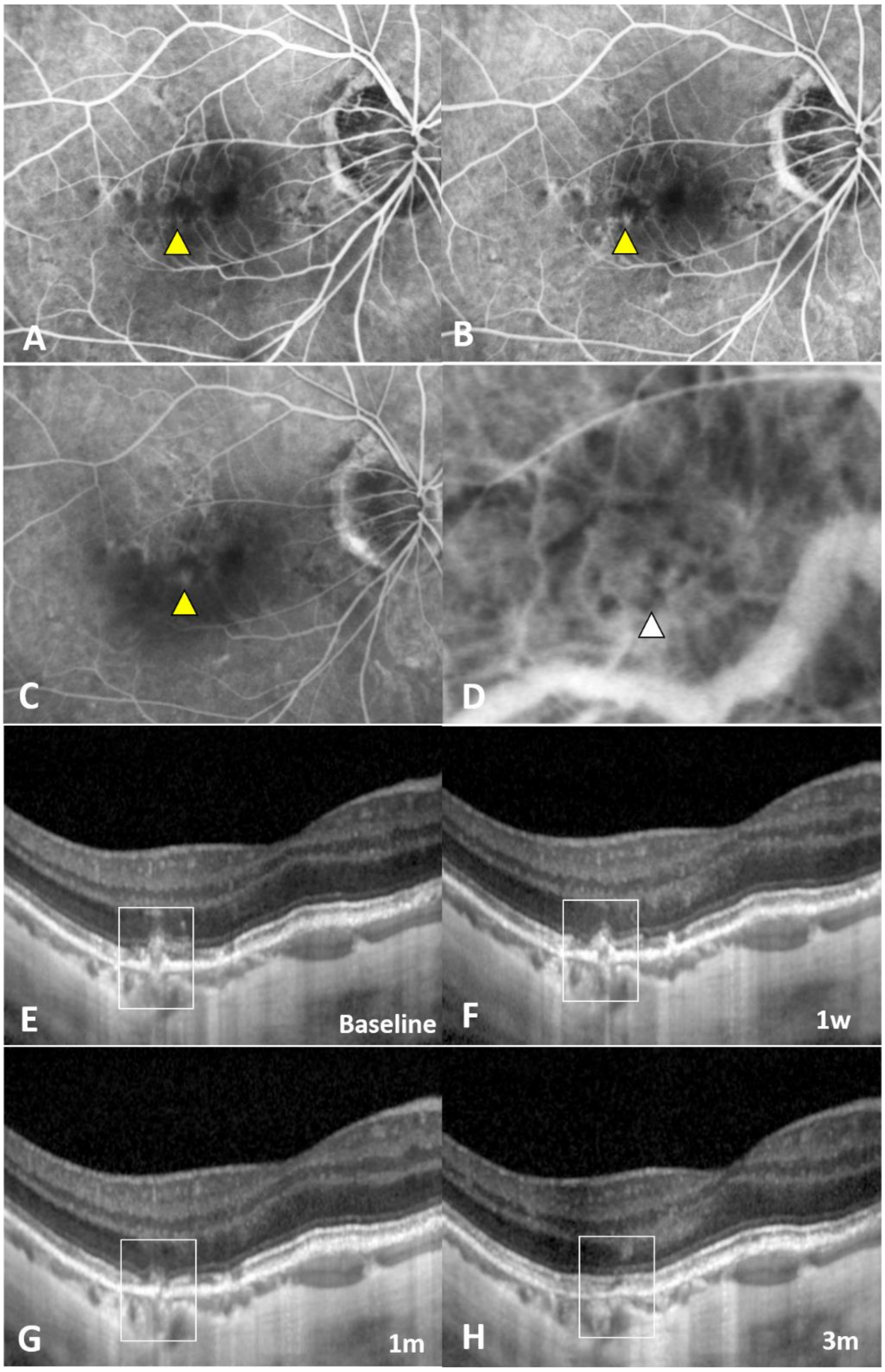

Multimodal imaging characteristics of a 50-year-old man with metamorphopsia in front of his right eye for 2 days (refractive error: -16.0D). **A-C**. Fluorescein angiography showing early hyperfluorescence with mild leakage (yellow arrowhead) during the late phase within the extrafoveal hemorrhage. **D.** Indocyaninegreen angiography showing linear hypofluorescence of a lacquer crack (white arrowhead). **E.** Spectral-domain optical coherence tomography showing a focal rupture of the RPE-BM-CC complex, without an exudative sign (white square). **F-H.** With absorption of the subretinal hemorrhage, the ruptured RPE-BM-CC complex gradually resolved during the 3-month follow up (white square).
